# Analgesic Effects of Fisetin, Peimine, Astaxanthin, Artemisinin, Bardoxolone Methyl and 740 Y-P and Their Influence on Opioid Analgesia in a Mouse Model of Neuropathic Pain

**DOI:** 10.3390/ijms24109000

**Published:** 2023-05-19

**Authors:** Katarzyna Ciapała, Ewelina Rojewska, Katarzyna Pawlik, Agata Ciechanowska, Joanna Mika

**Affiliations:** Department of Pain Pharmacology, Maj Institute of Pharmacology Polish Academy of Sciences, 12 Smetna Str., 31-343 Krakow, Poland; kat.ciapala@gmail.com (K.C.); rojewska@if-pan.krakow.pl (E.R.); pawlik@if-pan.krakow.pl (K.P.); ciechan@if-pan.krakow.pl (A.C.)

**Keywords:** analgesia, MAPK inhibitors, fisetin, peimine, astaxanthin, morphine, buprenorphine, oxycodone

## Abstract

Treatment of neuropathic pain remains a challenge for modern medicine due to the insufficiently understood molecular mechanisms of its development and maintenance. One of the most important cascades that modulate the nociceptive response is the family of mitogen-activated protein (MAP) kinases and phosphatidylinositol-3-kinase (PI3K), as well as nuclear factor erythroid 2-related factor 2 (Nrf2). The aim of this study was to determine the effect of nonselective modulators of MAP kinases—fisetin (ERK1/2 and NFκB inhibitor, PI3K activator), peimine (MAPK inhibitor), astaxanthin (MAPK inhibitor, Nrf2 activator) and artemisinin (MAPK inhibitor, NFκB activator), as well as bardoxolone methyl (selective activator of Nrf2) and 740 Y-P (selective activator of PI3K)—in mice with peripheral neuropathy and to compare their antinociceptive potency and examine their effect on analgesia induced by opioids. The study was performed using albino Swiss male mice that were exposed to chronic constriction injury of the sciatic nerve (CCI model). Tactile and thermal hypersensitivity was measured using von Frey and cold plate tests, respectively. Single doses of substances were administered intrathecally on day 7 after CCI. Among the tested substances, fisetin, peimine, and astaxanthin effectively diminished tactile and thermal hypersensitivity in mice after CCI, while artemisinin did not exhibit analgesic potency in this model of neuropathic pain. Additionally, both of the activators tested, bardoxolone methyl and 740 Y-P, also showed analgesic effects after intrathecal administration in mice exposed to CCI. In the case of astaxanthin and bardoxolone methyl, an increase in analgesia after combined administration with morphine, buprenorphine, and/or oxycodone was observed. Fisetin and peimine induced a similar effect on tactile hypersensitivity, where analgesia was enhanced after administration of morphine or oxycodone. In the case of 740 Y-P, the effects of combined administration with each opioid were observed only in the case of thermal hypersensitivity. The results of our research clearly indicate that substances that inhibit all three MAPKs provide pain relief and improve opioid effectiveness, especially if they additionally block NF-κB, such as peimine, inhibit NF-κB and activate PI3K, such as fisetin, or activate Nrf2, such as astaxanthin. In light of our research, Nrf2 activation appears to be particularly beneficial. The abovementioned substances bring promising results, and further research on them will broaden our knowledge regarding the mechanisms of neuropathy and perhaps contribute to the development of more effective therapy in the future.

## 1. Introduction

Neuropathic pain is a chronic condition with multifactorial, still not fully understood pathogenesis resulting from damage to somatosensory neurons in the peripheral and central nervous systems, and its treatment remains a considerable challenge for modern medicine [1]. According to statistics, this kind of pain affects approximately 7–10% of the population worldwide, and less than half of patients report actual pain relief [2]. Understanding the molecular mechanisms that underlie neuropathic pain is essential for the design of new therapeutic strategies. Even opioids, which are one of the strongest analgesic drugs, will not provide effective pain reduction for every patient with neuropathy, and their prolonged use is associated with the occurrence of adverse effects [3,4]. Nonetheless, these drugs can efficiently relieve moderate-to-severe chronic pain and are widely used in the clinic. Neuronal and glial cell activation are commonly considered critical in the progression of neuropathic pain [5,6,7], and these processes are associated with modifications to intracellular signaling pathways [8]. One of the most important cascades that modulates the nociceptive response is the mitogen-activated protein kinase (MAPK) family [9,10]. This evolutionally conserved group contains p38 mitogen-activated protein (p38) and extracellular-regulated (ERK) and c-Jun N-terminal (JNK) kinases. A multiplicity of extracellular stimuli trigger MAPK phosphorylation, leading to different posttranslational and transcriptional downstream responses [11]. Several studies have shown that all three MAPKs are involved in the modulation of nociceptive information, as well as central sensitization [12,13,14,15,16]. A variety of selective MAPK inhibitors, e.g., SB203580, D-JNKI-1, SP600125 and PD198306, have been described to significantly attenuate tactile and thermal hyperalgesia [15,17,18]. However, more effective substances that could have a wider spectrum of action are still being sought. Another interesting target is phosphatidylinositol-3-kinase (PI3K), which is a lipid kinase widely expressed in the spinal cord, particularly in the dorsal horn, where nociceptive C and Aδ fibers of primary afferents principally terminate [19]. Recently, PI3K and its downstream signaling were shown to regulate hypersensitivity [20]. Moreover, at the spinal cord level, the nuclear factor kappa-light-chain-enhancer of activated B cells (NF-κB) seems to be another important pharmacological target for neuropathic pain relief, since its activation induces the expression of many nociceptive molecules [21,22,23]. It has already been proven that selective NF-κB inhibitors (such as PDTC or SN50) diminish neuropathic pain [24,25]. Accumulating evidence suggests that among transcription factors, nuclear factor erythroid 2-related factor 2 (Nrf2), which regulates antioxidant defense, might also be important for pharmacological treatment. In 2021, it was shown that Nrf2 and its downstream targets appear to be involved in neuropathic pain [26]. In summary, the available literature suggests that changes in p38, ERK1/2, NF-κB, Nrf2 and PI3K play an important, but not fully understood role in the pathogenesis of neuropathic pain and that they may contribute to the downstream activation of many nociceptive factors; therefore, they became the subject of our research. Moreover, new nonspecific bioactive compounds are currently receiving attention, which is why in our research, we tested mainly substances with a broad spectrum of action and some that are safe for people and already approved as dietary supplements. However, their analgesic properties have not been clearly proven thus far.

Therefore, the aim of this study was to examine the influence of different nonselective MAPK inhibitors, such as fisetin (ERK1/2 and NFκB inhibitor, PI3K activator) [27,28,29,30], peimine (MAPK and NFκB inhibitor) [31], astaxanthin (MAPK inhibitor, Nrf2 activator) [32,33,34,35] and artemisinin (MAPK inhibitor, NFκB activator) [36,37,38], on tactile and thermal hypersensitivity on day 7 after chronic constriction injury (CCI) of the sciatic nerve (neuropathic pain model) in mice. Due to the lack of published data, for comparison, we also studied the effectiveness of a selective activator of Nrf2 (bardoxolone methyl) [39,40] and a selective activator of PI3K (740 Y-P) [41] in CCI-evoked neuropathy. Third, we determined the ability of these modulators to improve the analgesic properties of morphine, buprenorphine and oxycodone in neuropathic pain, especially since all three MAPK and PI3K signaling pathways have been reported to be involved in opioid effectiveness [42,43].

## 2. Results

### 2.1. The effect of a Single i.t. Administration of Artemisinin, Peimine, Fisetin and Astaxanthin on Tactile Hypersensitivity on the 7th Day after CCI

**Figure 1:**
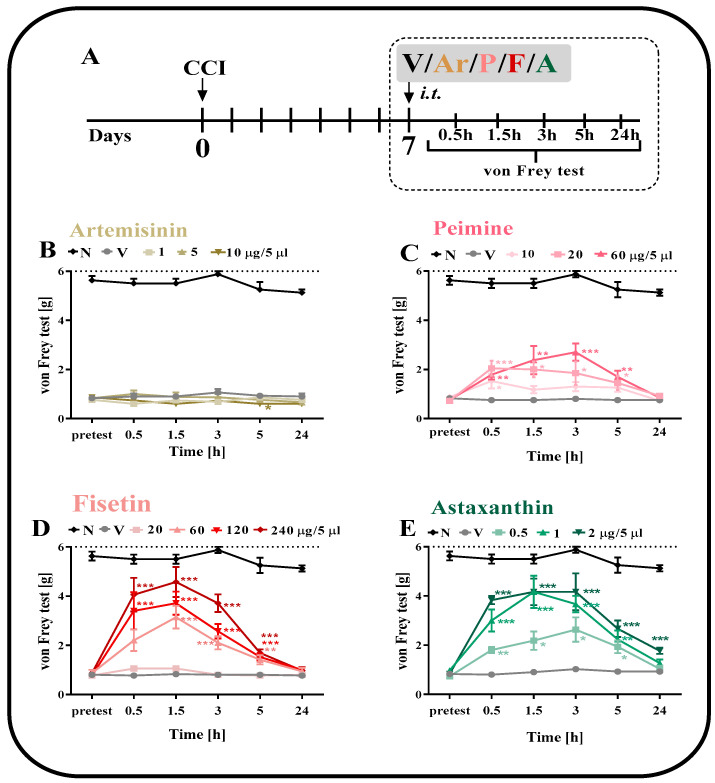
Pharmacological modulation by nonselective, mitogen-activated protein kinase inhibitors influences tactile hypersensitivity (**A**–**E**) in a neuropathic pain model. The effect of single intrathecal administrations of artemisinin (1, 5, 10 µg/5 µL, (**B**), peimine (10, 20, 60 µg/5 µL, (**C**), fisetin (20, 60, 120, 240 µg/5 µL, (**D**) or astaxanthin (0.5, 1, 2 µg/5 µL (**E**) on mechanical stimuli was assessed at 0.5, 1.5, 3, 5 and 24 h after treatment on the 7th day post-CCI using the von Frey test (**A**). The data are presented as means ± SEMs (V n = 8; Ar n = 6; P n = 8; F n = 7; A n = 6—each group). The horizontal dotted line shows the cutoff values. The intergroup differences were analyzed using ANOVA with Bonferroni’s multiple comparisons test. * *p* < 0.05, ** *p* < 0.01 and *** *p* < 0.001 indicate differences between V-treated CCI-exposed mice and substance-treated CCI-exposed mice. Abbreviations: Ar, artemisinin; P, peimine; F, fisetin; A, astaxanthin; CCI, chronic constriction injury; N, naïve; V, vehicle.

Single i.t. injections of intracellular pathway modulators were administered to CCI-exposed mice on day 7 after surgery (Figure 1A). Artemisinin did not influence tactile hypersensitivity measured with the von Frey test compared to vehicle-treated mice at any point after treatment (Figure 1B). In contrast, peimine, fisetin and astaxanthin dose-dependently diminished tactile hypersensitivity on day 7 after CCI.

The most effective dose of peimine was 60 µg/5 µL, which had significantly reduced tactile hypersensitivity 3 h after injection (F_(3, 28)_ = 6653; *p* = 0.0016), while a dose of 10 µg/5 µL was observed to be effective only 0.5 h after administration (Figure 1C). In the case of fisetin, doses of 60, 120 and 240 µg/5 µL evoked analgesic effects that peaked 1.5 h after administration (F_(4, 30)_ = 16.62; *p* < 0.0001), while the lowest dose (20 µg/5 µL) did not cause analgesia at any studied time point (Figure 1D). Astaxanthin dose-dependently caused a decrease in tactile hypersensitivity for each dose used, and after 24 h, this effect was no longer observed, except for the highest dose (2 µg/5 µL), which was still significantly effective (F_(3, 22)_ = 9490; *p* = 0.0003) (Figure 1E). Two-way ANOVA confirmed significant interaction between the investigated treatment and the subjected time points for fisetin (F_(20, 178)_ = 6026; *p* < 0.0001), peimine (F_(15, 168)_ = 2556; *p* = 0.0019) and astaxanthin (F_(15, 132)_ = 5238; *p* < 0.0001).

### 2.2. The Effect of a Single i.t. Administration of Artemisinin, Peimine, Fisetin and Astaxanthin on Thermal Hypersensitivity on the 7th Day after CCI

**Figure 2:**
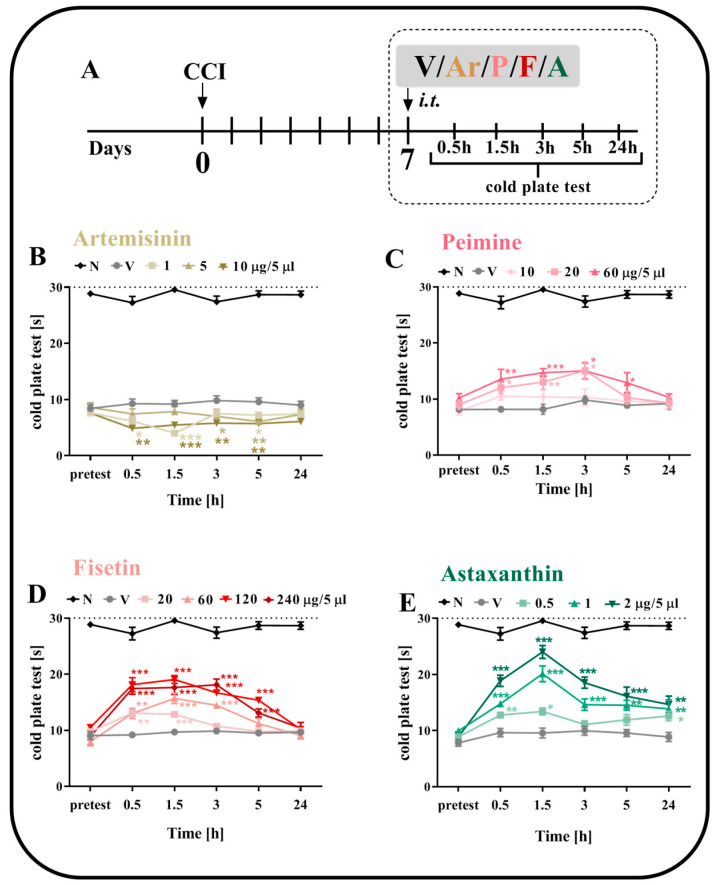
Pharmacological modulation by nonselective, mitogen-activated protein kinase inhibitors influences thermal hypersensitivity (**A**–**E**) in a neuropathic pain model. The effect of single intrathecal administrations of artemisinin (1, 5, 10 µg/5 µL, (**B**), peimine (10, 20, 60 µg/5 µL, (**C**), fisetin (20, 60, 120, 240 µg/5 µL, (**D**) or astaxanthin (0.5, 1, 2 µg/5 µL, (**E**) on thermal hypersensitivity was assessed at 0.5, 1.5, 3, 5 and 24 h after treatment on the 7th day post-CCI with the cold plate test (**A**). The data are presented as means ± SEMs (V n = 8; Ar n = 6; P n = 8; F n = 7; A n = 6—each group). The horizontal dotted line shows the cutoff values. The intergroup differences were analyzed using ANOVA with Bonferroni’s multiple comparisons test. * *p* < 0.05, ** *p* < 0.01 and *** *p* < 0.001 indicate differences between V-treated CCI-exposed mice and substance-treated CCI-exposed mice. Abbreviations: Ar, artemisinin; P, peimine; F, fisetin; A, astaxanthin; CCI, chronic constriction injury; N, naïve; V, vehicle.

Single i.t. injections of intracellular pathway modulators were administered to CCI-exposed mice on day 7 after surgery (Figure 2A).

Surprisingly, a single administration of artemisinin enhanced thermal hypersensitivity measured with the cold plate test compared to vehicle-treated mice (F_(3, 20)_ = 5740; *p* = 0.0053); however, 24 h after administration, this effect was no longer observed (Figure 2B). In contrast, peimine (20, 60 µg/5 µL), fisetin (20, 60, 120, 240 µg/5 µL) and astaxanthin (0.5, 1, 2 µg/5 µL) effectively diminished pain-related behavior. Peimine at the lowest dose (10 µg/5 µL) did not influence thermal hypersensitivity at any tested time point (Figure 2C). Regarding fisetin, each dose evoked an analgesic effect, which lasted up to 5 h after administration in the case of doses of 120 and 240 µg/5 µL (F_(4, 28)_ = 13.62; *p* < 0.0001) (Figure 2D). The most effective reduction of thermal hypersensitivity of astaxanthin was observed for the dose of 2 µg/5 µL 1.5 h after administration (F_(3, 22)_= 40.49; *p* < 0.0001). This substance caused a decrease in thermal hypersensitivity at each dose used, which lasted up to 24 h (F_(3, 22)_ = 5110; *p* = 0.0078) (Figure 2E). Two-way ANOVA confirmed interaction between the investigated treatment and the subjected time points in case of fisetin (F_(20, 173)_ = 6; *p* < 0.0001) and astaxanthin (F_(15, 132)_ = 4; *p* < 0.0001).

### 2.3. The Effect of a Single i.t. Administration of Bardoxolone Methyl or 740 Y-P on Pain-Related Behaviors on Day 7 after CCI

**Figure 3:**
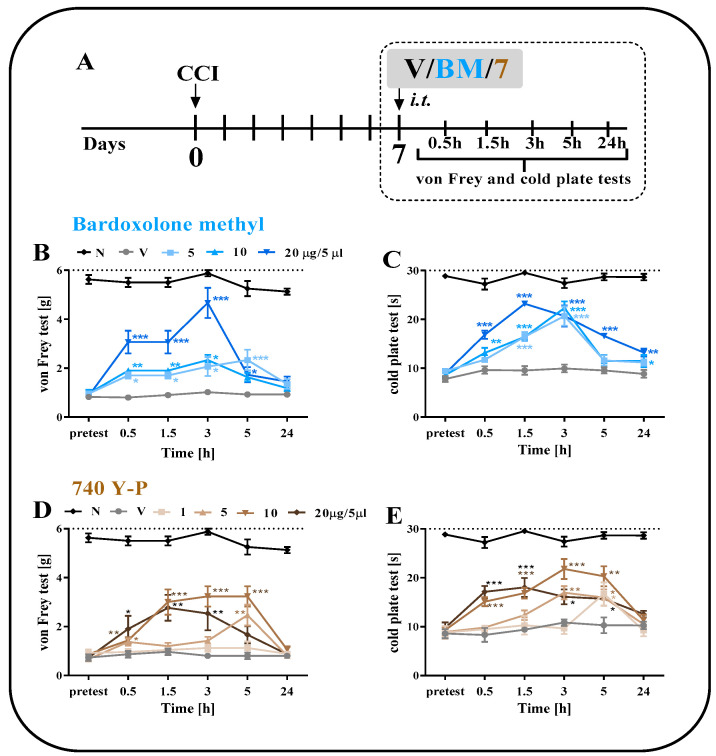
Pharmacological modulation by Nrf2 or Pi3K activators influences tactile and thermal hypersensitivity (**A**–**E**) in a neuropathic pain model. The effect of single intrathecal administrations of bardoxolone methyl (5, 10, 20 µg/5 µL (**B**,**C**)) or 740 Y-P (1, 5, 10, 20 µg/5 µL (**D**,**E**)) on tactile and thermal hypersensitivity was assessed at 0.5, 1.5, 3, 5 and 24 h after treatment on the 7th day post-CCI with von Frey (**B**,**D**) and cold plate (**C**,**E**) tests. The data are presented as means ± SEMs (V n = 8; BM n = 6; 740 Y-P n = 6—each group except the lowest dose of 740 Y-P n = 5). The horizontal dotted line shows the cutoff values. The intergroup differences were analyzed using ANOVA with Bonferroni’s multiple comparisons test. * *p* < 0.05, ** *p* < 0.01 and *** *p* < 0.001 indicate differences between V-treated CCI-exposed mice and substance-treated CCI-exposed mice. Abbreviations: BM, bardoxolone methyl; 7, 740 Y-P; V, vehicle; CCI, chronic constriction injury; N, naïve; V, vehicle.

A single i.t. injection of bardoxolone methyl diminished tactile hypersensitivity measured using the von Frey test compared to vehicle-treated mice at 0.5, 1.5, 3 and 5 h after treatment (Figure 3A,B). The most effective dose was 20 µg/5 µL, which(F_(3, 22)_ = 19.31; *p* < 0.0001) attenuated tactile hypersensitivity 3 h after injection with the greatest potency (Figure 3B).

Regarding the cold plate test, each dose of bardoxolone methyl caused a similarly good analgesic effect 3 h after administration (F_(3, 22)_ = 15,19; *p* < 0.0001) (Figure 3A,C). The doses of 5 and 10 µg/5 µL produced similar attenuation of tactile and thermal hypersensitivity on the von Frey (Figure 3B) and cold plate (Figure 3C) tests. A reduction in tactile hypersensitivity was no longer observed after 24 h, while the decrease in thermal hypersensitivity persisted until 24 h for doses of 10 and 20 µg/5 µL. Two-way ANOVA confirmed significant interaction between the investigated treatment and the subjected time points in von Frey (F_(15, 132)_ = 6856; *p* < 0.0001) and cold plate (F_(15, 130)_ = 6610; *p* < 0.0001) tests.

A single i.t. injection of 740 Y-P (1, 5, 10 and 20 µg/5 µL) diminished tactile (Figure 3A,D) and thermal (Figure 3A,E) hypersensitivity compared to vehicle-treated mice. The dose of 1 µg/5 µL was ineffective in the von Frey test, but in the cold plate test, it exerted a slight analgesic effect, but only 5 h after administration (Figure 3E) (F_(4, 24)_ = 4048; *p* = 0.0120). Similarly, doses of 10 and 20 µg/5 µL most effectively attenuated CCI-induced tactile (Figure 3D) and thermal (Figure 3E) hypersensitivity. After 24 h, these effects were not observed in any of the tests. Two-way ANOVA confirmed significant interaction between the investigated treatment and the subjected time points in von Frey (F_(20, 144)_ = 3708; *p* < 0.0001) and cold plate (F_(20, 144)_ = 3321; *p* < 0.0001) tests.

### 2.4. Comparison of the Analgesic Effects of a Single i.t. Administration of Artemisinin, Fisetin, Peimine, Astaxanthin, Bardoxolone Methyl or 740 Y-P on Day 7 after CCI

According to the von Frey test, astaxanthin and 740 Y-P are the substances with the lowest ED_50_ value, while the highest dose of peimine is required to obtain significant pain relief compared to other substances. On the cold plate test, astaxanthin had the lowest ED_50_ value, while peimine had the highest ED_50_ value. It was not possible to determine this value for artemisinin, since this substance after i.t. injection did not exert analgesic effects (Table 1).

Regarding the %MPE calculated for the highest doses of tested substances in von Frey and cold plate tests, fisetin, astaxanthin and bardoxolone methyl were the compounds that produced the most significant antinociception (Table 2). In contrast, artemisinin was ineffective in both behavioral tests.

### 2.5. The Effect of a Single i.t. Administration of Peimine, Fisetin and Astaxanthin on Opioid Effectiveness on the 7th Day after CCI

**Figure 4:**
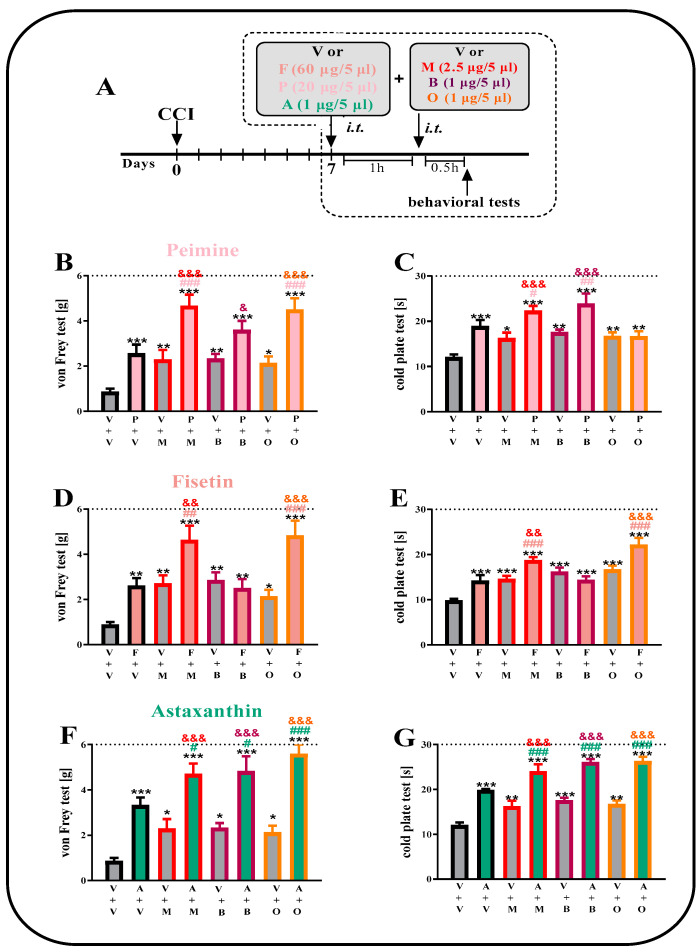
Pharmacological modulation by nonselective, mitogen-activated protein kinase inhibitors influences opioid effectiveness (**A**–**G**) in a neuropathic pain model. After 1 h, the single vehicle-, peimine- (20 µg/5 µL), fisetin- (60 µg/5 µL) or astaxanthin- (1 µg/5 µL)-treated mice received a single dose of vehicle, morphine (2.5 µg/5 µL), buprenorphine (1 µg/5 µL) or oxycodone (1 µg/5 µL), and von Frey (**B**,**D**,**F**) and cold plate (**C**,**G**,**E**) tests were conducted 30 min later. The data are presented as means ± SEMs (V + V n = 6–7; P + V n = 6; V + M n = 5–7; P + M n = 6; V + B n = 6–7; P + B n = 5; V + O n = 6–7; P + O n = 6; F + V n = 6–7; F + M n = 7; F + B n = 7; F + O n = 6; A + V n = 6; A + M n = 7; A + B n = 6; A + O n = 5). The horizontal dotted line shows the cutoff values. The results were analyzed using one-way ANOVA with Bonferroni’s multiple comparisons post hoc test. * *p* < 0.05, ** *p* < 0.01, and *** *p* < 0.001 indicate differences compared with V + V-treated CCI-exposed mice; ^&^ *p* < 0.05, ^&&^ *p* < 0.01 and ^&&&^ *p* < 0.001 indicate differences between the V + M/B/O- and P/F/A + M/B/O-treated CCI-exposed mice; and ^#^ *p* < 0.05, ^##^ *p* < 0.01 and ^###^ *p* < 0.001 indicates differences between the P/F/A + V- and P/F/A + M/B/O-treated CCI-exposed mice. Abbreviations: Ar, artemisinin; P, peimine; F, fisetin; A, astaxanthin; M, morphine; B, buprenorphine; O, oxycodone; V, vehicle; CCI, chronic constriction injury.

Since artemisinin did not exert analgesic effects (Figure 1B and Figure 2B), peimine, fisetin and astaxanthin were used (Figure 4A) to test their influence on opioid analgesia, and doses for the following experiments were selected based on a dose-dependence study. Single i.t. injections of morphine (2.5 µg/5 µL), buprenorphine (1 µg/5 µL) or oxycodone (1 µg/5 µL) evoked similar levels of analgesia on von Frey (Figure 4B,D,F) and cold plate (Figure 4C,E,G) tests. 

Single i.t. administration of peimine at a dose of 20 µg/5 µL significantly attenuated CCI-induced tactile and thermal hypersensitivity on von Frey (F_(7, 40)_ = 15.18; *p* < 0.0001) and cold plate (F_(7, 38)_ = 11.72; *p* < 0.0001) tests compared to the vehicle-treated group (Figure 4B,C). The administration of peimine with opioids resulted in more effective attenuation of tactile hypersensitivity in the case of morphine and oxycodone, while thermal hypersensitivity was more effectively reduced in the case of administration with morphine or buprenorphine.

Single i.t. administration of fisetin (60 µg/5 µL) resulted in a decrease in tactile (F_(7, 46)_= 9284; *p* < 0.0001) and thermal (F_(7, 45)_= 16.31; *p* < 0.0001) hypersensitivity (Figure 4D,E). The administration of fisetin with opioids resulted in more effective attenuation of tactile and thermal hypersensitivity in the case of morphine and oxycodone, while it did not influence buprenorphine analgesia (Figure 4D,E).

Astaxanthin administered i.t. (1 µg/5 µL) exerted an analgesic effect, and importantly, injection of this substance with morphine, buprenorphine or oxycodone resulted in a greater decrease in tactile (F_(7, 40)_ = 16.58; *p* < 0.0001) (Figure 4F) and thermal (F_(7, 39)_ = 29.54; *p* < 0.0001) (Figure 4G) hypersensitivity compared to each of these substances administered alone.

### 2.6. The Effect of a Single i.t. Administration of Bardoxolone Methyl or 740 Y-P on Opioid Effectiveness on the 7th Day after CCI

**Figure 5:**
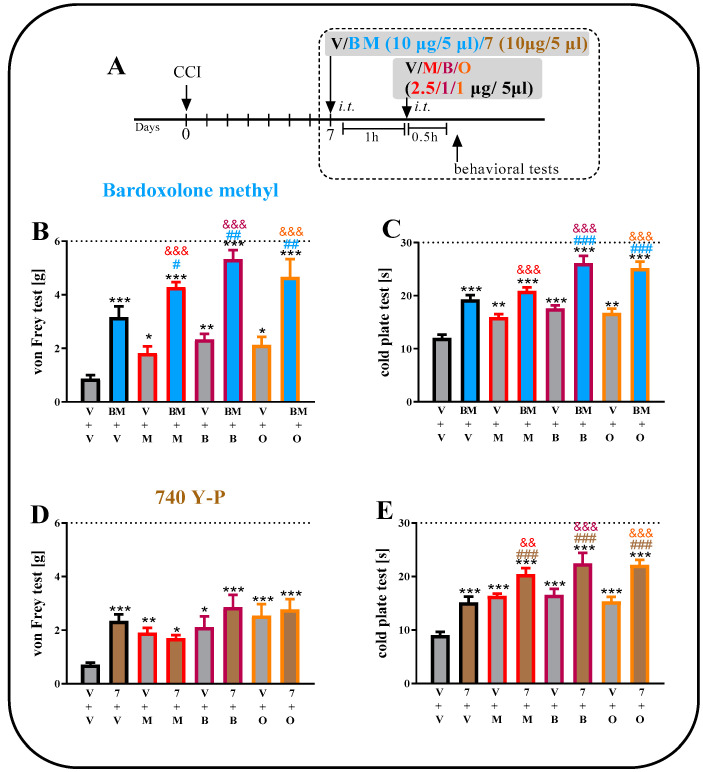
Pharmacological modulation by Nrf2 and Pi3K activators influences opioid effectiveness (**A**–**E**) in a neuropathic pain model. After 1 h, the single vehicle-, bardoxolone methyl- (10 µg/5 µL; (**B**,**C**)) or 740 Y-P- (10 µg/5 µL; (**D**,**E**))-treated mice received a single dose of vehicle, morphine (2.5 µg/5 µL), buprenorphine (1 µg/5 µL) or oxycodone (1 µg/5 µL), and von Frey (**B**,**D**) and cold plate (**C**,**E**) tests were conducted 30 min later. The data are presented as means ± SEMs (V + V n = 6–7; BM + V n = 6–7; V + M n = 6–8; BM + M n = 7; V + B n = 6–7; BM + B n = 6; V + O n = 6–7; BM + O n = 6; 7 + V n = 7; 7 + M n = 8; 7 + B n = 7; 7 + O n = 7). The horizontal dotted line shows the cutoff values. The results were analyzed using one-way ANOVA with Bonferroni’s multiple comparisons post hoc test. * *p* < 0.05, ** *p* < 0.01, and *** *p* < 0.001 indicate differences compared with V + V-treated CCI-exposed mice; ^&&^ *p* < 0.01 and ^&&&^ *p* < 0.001 indicate differences between the V + M/B/O- and BM/7 + M/B/O-treated CCI-exposed mice; and ^#^ *p* < 0.05, ^##^ *p* < 0.01 and ^###^ *p* < 0.001 indicate differences between the BM/7 + V- and BM/7 + M/B/O-treated CCI-exposed mice. Abbreviations: BM, bardoxolone methyl; 7, 740 Y-P; B, buprenorphine; M, morphine; O, oxycodone; V, vehicle; CCI, chronic constriction injury.

The single i.t. administration of bardoxolone methyl (10 µg/5 µL) significantly attenuated CCI-induced tactile and thermal hypersensitivity on von Frey and cold plate tests compared to the vehicle-treated group (Figure 5A–C). Similarly, single i.t. administration of 740 Y-P (10 µg/5 µL) evoked a decrease in hypersensitivity to tactile (Figure 5A,D) and thermal stimuli (Figure 5A,E). Additionally, single i.t. injection of morphine(2.5 μg/5 μL), buprenorphine (1 μg/5 μL) or oxycodone (1 μg/5 μL) caused analgesic effects on the von Frey (Figure 5B,D) and cold plate (Figure 5C,E) tests.

The administration of morphine, buprenorphine or oxycodone preceded by injection of bardoxolone methyl resulted in a more effective attenuation of tactile (F_(7, 40)_ = 12.13; *p* < 0.0001) (Figure 5B) and thermal (F_(7, 39)_ = 23,70; *p* < 0.0001) (Figure 5C) hypersensitivity. The injection of all tested opioids preceded by treatment with 740 Y-P resulted in a more effective attenuation of thermal (F_(7, 48)_ = 11,85; *p* < 0.0001) hypersensitivity (Figure 5E), but had no influence on tactile hypersensitivity (Figure 5D).

## 3. Discussion

**Scheme 1:**
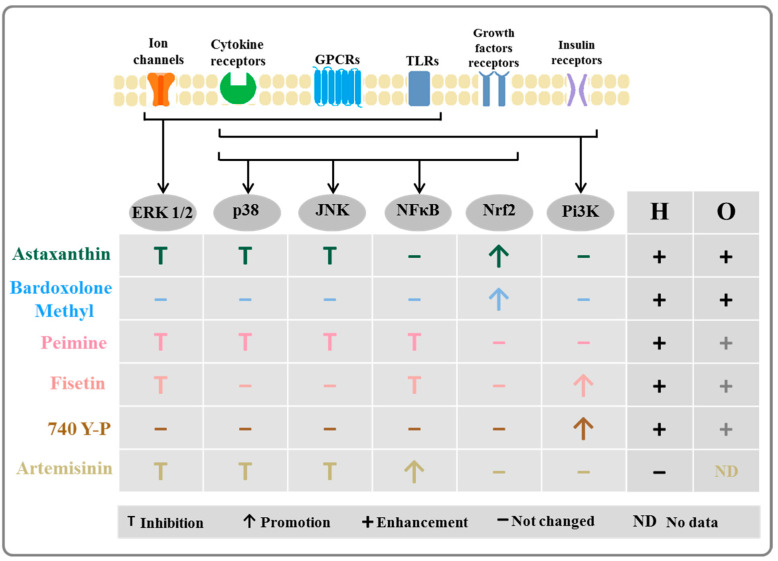
Modulators of intracellular kinases and transcription factors and their influence on pain hypersensitivity (H) and opioid analgesia (O) in mice exposed to CCI.

This study showed that among the nonselective MAPK modulators, fisetin, peimine and astaxanthin effectively diminished tactile and thermal hypersensitivity in mice after nerve injury, while artemisinin did not exert analgesic potency in the model of neuropathic pain used (Scheme 1).

For the first time, both of the activators tested, bardoxolone methyl and 740 Y-P, also showed analgesic effects after intrathecal administration in mice exposed to sciatic nerve injury. The results of our research clearly indicate that substances that inhibit all three MAPKs provide pain relief, especially if they additionally block NF-κB, such as peimine, activate PI3K, such as fisetin, and activate Nrf2, such as astaxanthin, the most effective substance in our research (Scheme 1).

Considering that some of the substances used have the status of dietary supplements, further research, both experimental and clinical, in light of such promising results, is undoubtedly recommended. Importantly, the results of our research indicate that substances such as peimine, fisetin, bardoxolone methyl and especially astaxanthin increase the effectiveness of morphine, buprenorphine and/or oxycodone, which is an exceptionally important outcome from a clinical point of view. For our experiments, we selected substances that modulate intracellular pathways that are considered to participate in the development of hypersensitivity in neuropathy. Activation of p38 is regarded as crucial step in the development of neuroinflammation, and a growing amount of evidence has shown increased activation of this kinase at different levels of nociceptive pathways in various pain models [12,44,45,46,47]. Importantly, some experiments, including those performed by our group, revealed that intrathecal injection of selective inhibitors of p38, such as SB203580 [12,48,49] or FR167653 [50], diminished pain-related behavior in models of neuropathic pain. Notably, the first clinical trials were undertaken to assess the analgesic action of the potent p38 inhibitors dilmapimod (SB-681323) [51] and losmapimod (GW856553) [52] in patients suffering from neuropathic pain. The first appears to be promising; however, it merits further evaluation in larger clinical trials, particularly to estimate the size of analgesic effect. However, recently, experimental studies have provided evidence that nonselective substances with a broad spectrum of action appear to have great therapeutic potential, and one of them is minocycline, which, apart from being a p38 inhibitor, also impairs ERK1/2 and JNK signaling [53,54]. To date, minocycline has been shown to reduce hypersensitivity in nerve injury- [55,56,57] and diabetic- [58]-evoked neuropathy and has become a promising therapeutic drug in the clinic [59]. ERK signaling is linked to potassium channel phosphorylation and changes in voltage-gated calcium channels of sensory neurons [59]. This was confirmed by pharmacological studies that showed that selective inhibitors of the ERK pathway, such as PD198306 [60], U0126 [15,61] and PD98059 [18,62], diminished pain-related behavior in models of neuropathy. In contrast to p38, which is mainly expressed in microglia/macrophages after nerve injury, JNK changes were detected in neurons and astrocytes [63,64]. Importantly, it was also proven that injection of the selective JNK inhibitors D-JNKI-1JNK or SP600125 prevented hypersensitivity in animals with neuropathic pain [65,66]. Since selective p38, ERK1/2 or JNK inhibitors have been shown to be effective in relieving neuropathic pain in animal models, we hypothesize that substances acting on the entire MAPK family and administered directly at the spinal cord level could be even more effective.

To our surprise, artemisinin, a sweet wormwood-derived compound that is considered to be an all-MAPK inhibitor [36,37], did not exert analgesic potency in CCI-exposed mice when administered intrathecally. In contrast, experiments performed by others indicate that this substance has analgesic potency in neuropathic [67] and inflammatory [68,69] pain, but only after repeated intraperitoneal administration, which suggests its divergent molecular mechanisms of action on the periphery. However, published data indicate that this compound additionally acts on the transcription factor NF-κB; however, some in vitro studies point to inhibitory [37] and others to stimulatory [38] effects. The lack of effectiveness of artemisinin in our studies suggests that its i.t. administration may cause unfavorable NF-κB activation in neuropathy; however, this requires additional investigation. To date, it is known that NF-κB plays an important role in nociceptive transmission since it regulates the synthesis of many pronociceptive factors that are upregulated after nerve injury [70,71,72]. Our previous results obtained by Western blot analysis have shown that after CCI in rats, the level of NF-κB is increased [18,73] and its inhibition by parthenolide diminishes hypersensitivity [15,73]. This is a possible explanation for why we obtained such good analgesic effects after i.t. administration of peimine, which is an inhibitor of all three MAPKs and NF-κB. We showed for the first time that this substance strongly reduces hypersensitivity to mechanical and thermal stimuli in mice exposed to CCI. The biological properties of peimine are still being explored, although its health-promoting effect has been known for a long time. The compound has been isolated from *Fritillaria*, a plant used in traditional Chinese medicine as an anti-inflammatory and analgesic remedy [74]; however, it is not yet recommended for therapy in Europe or the USA. Importantly, recent research shows that apart from blocking MAPK and NF-κB, peimine blocks the Nav1.7-gated sodium and Kv1.3 potassium channels [74], which might also contribute to its analgesic properties. Taking into account published data and the results obtained by us, we believe that peimine could be considered in the treatment of neuropathy, especially because it was recently pointed out that this substance targets nAChRs with high affinity, which might account for its anti-inflammatory actions [75]. Undoubtedly, further studies are necessary since preclinical and clinical outcomes are very promising.

Another very interesting substance that modulates MAPK and inhibits NF-κB is fisetin, a naturally occurring flavonoid [27]. In our studies, we have shown that this compound has an analgesic effect in mice subjected to CCI, even after a single injection. In 2015, it was proven that repeated fisetin treatment exerts an antinociceptive effect on thermal hypersensitivity in CCI-exposed mice [76]. It was also demonstrated by others that fisetin administration can delay diabetic neuropathic pain-related behavior in mice [77]. Importantly, fisetin also influences the PI3K/AKT/mTOR pathway, which is unique for its multiplicity of roles in the transcriptional regulation of genes important in the development of neuropathic pain. The latest research indicates that this substance acts as a PI3K activator [30,78]. Interestingly, in 2019, it was proven that resveratrol, another plant-derived compound, alleviated paclitaxel-induced neuropathic pain through activation of the PI3K/Akt pathway [79]. Therefore, to address these findings, in our study, we decided to use a selective PI3K activator, namely, 740 Y-P, and for the first time, we showed that this substance diminished tactile and thermal hypersensitivity in CCI-exposed mice. This interesting result sheds new light on testing the effectiveness of substances that are activators of intracellular cascades in neuropathic pain.

Among the substances we tested, astaxanthin exerted the best analgesic effect in the CCI model of neuropathic pain. This is also indicated by the analysis of the ED_50_ values for each of the substances tested, according to which astaxanthin is effective at lower doses than the other substances (Table 1). Astaxanthin is a carotenoid found primarily in various plants, algae and seafood, and it exerts antioxidant, anti-inflammatory and antiapoptotic properties [80]. It was previously hypothesized that astaxanthin could relieve neuropathic pain by spinal inhibition of ERK1/2, p38 and NF-κB activation in a spinal nerve ligation model [81]. Data from in vitro studies suggest that this substance reduces the oxidative stress induced by lipopolysaccharides in C6 glial cells [82]. Moreover, it was proposed that astaxanthin regulates IL-6 production through an ERK1/2-MSK-1- and NF-κB p65-dependent pathway [83]. Interestingly, a subsequent study indicated that CCI surgery evoked an increase in kynurenine (KYN)/tryptophan (TRY), a decrease in serotonin (5-HT)/TRY and a decrease in the 5-HT/5-HIAA ratio in the spinal cord, which was reversed by chronic astaxanthin treatment [84]. Additionally, astaxanthin decreased thermal hypersensitivity and attenuated comorbid depressive-like behaviors in CCI-exposed mice [84]. Recently, another intracellular molecule was also proposed as a target for astaxanthin—Nrf2 [85]. This transcription factor plays a role in maintaining redox status, inflammatory modulation, protein degeneration and biotransformation. According to the latest literature, Nrf2 downstream interactions lead to the transcription of several antioxidative enzymes that can ameliorate neuropathic pain in rodent models [86,87,88]. The newest studies indicate that the antinociceptive effects of Nrf2 occur by reducing oxidative stress, neuroinflammation, and mitochondrial dysfunction. Interestingly, in our experiments, bardoxolone methyl, which is a selective activator of Nrf2, reduced tactile and thermal hypersensitivity in mice exposed to CCI. These results correspond well with a study by others, which also pointed out the beneficial action of bardoxolone methyl in ischemic optic neuropathy and in diabetic neuropathy [89,90].

In recent years, much attention has been given to the importance of polytherapy in the treatment of neuropathic pain, which is why we decided to verify whether the nonselective inhibitors and selective activators used in our studies can improve the effectiveness of opioid drugs in neuropathy. Opioids are considered to be a second- or third-line class of analgesics that may provide relief to some patients with chronic pain; however, in neuropathy, their analgesic potential significantly weakens [91,92]. Out of all the substances tested in this study, the best results were obtained in the case of astaxanthin, which, after a single administration with morphine, buprenorphine or oxycodone, evoked better analgesic effects than each of these drugs alone in CCI-exposed mice. Importantly, for the first time, we have shown that a substance that is an inhibitor of all three MAPK kinases and an activator of Nrf2 has such spectacular effects. However, this is consistent with previously published literature that confirmed the involvement of MAPKs in the analgesic potency of opioids. It was shown that inhibition of p38 [93], ERK1/2 [15,18] and JNK [94,95] improves the analgesic effects of morphine in neuropathy. It has also been proven that PD98059 (MEK 1/2 inhibitor) enhances buprenorphine-induced analgesia under neuropathic pain [18]; however, there is a lack of studies addressing oxycodone effectiveness. The action of astaxanthin is intriguing also because this substance is an activator of Nrf2. The latest published data indicate that Nrf2 activators, i.e., 5-fluoro-2-oxindole [96] and sulforaphane [97] increase the effectiveness of morphine in neuropathy. Our research shows, for the first time, that the coadministration of a selective activator of Nrf2, bardoxolone methyl with morphine, buprenorphine or oxycodone, evokes better analgesic effects than opioids alone on the von Frey test, but not in the case of morphine on the cold plate test, which needs to be explained in the future. Another very interesting substance with strong analgesic potential is peimine, which inhibits MAPKs and NF-κB. In our previous studies, we demonstrated that intrathecal injections of parthenolide (NF-κB inhibitor) significantly potentiate morphine analgesia in neuropathy [15,73]. Importantly, coinjection of peimine with opioids causes better attenuation of tactile hypersensitivity; however, we did not observe such effects for oxycodone in the case of thermal hypersensitivity, which remains to be clarified in additional experiments. Another tested substance, fisetin, which blocks ERK1/2 and is also an inhibitor of NF-κB and an activator of PI3K, improved the analgesic effects of morphine and oxycodone in both tests; however, it did not influence buprenorphine action. The possible explanation of this may be due to the different mechanisms of action of these opioids: morphine and oxycodone are strong MOR agonists [98,99], while buprenorphine is a MOR partial agonist, DOR and KOR antagonist and NOR strong agonist [100]. The reason that fisetin has a different effect on buprenorphine analgesia requires further research, but it might be related to the fact that it is a PI3K activator, and our experiments indicate that a selective PI3K activator did not influence the analgesic effectiveness of any of the opioids used on the von Frey test but evoked better analgesic effects on the cold plate test when coadministered with these opioids. This very interesting result might suggest that PI3K activation might have greater importance for the functioning of Aδ and C fibers, which are responsible for thermal sensation [101], with less impact on Aβ fibers, which are responsible for tactile hypersensitivity [102]. Additionally, published data indicate that rather than activation, the blockade of PI3K attenuated the development of morphine tolerance in mice with neuropathy [95,103]. Therefore, more studies are needed to explain the role of PI3K in neuropathic pain and opioid effectiveness. Importantly, our research shows that although the selective PI3K activator effectively relieves pain, it does not increase opioid analgesia.

## 4. Materials and Methods

### 4.1. Animals

Male albino Swiss mice (20–22 g) were purchased from Charles River (Hamburg, Germany) and housed in cages with sawdust bedding on a standard 12 h/12 h light/dark cycle (lights on at 06.00 a.m.), with food and water available ad libitum. All of the procedures were performed in accordance with the recommendations of the International Association for the Study of Pain (IASP) [104] and the National Institutes of Health (NIH) Guide for the Care and Use of Laboratory Animals and were approved by the Ethics Committee of the Maj Institute of Pharmacology, Polish Academy of Sciences (LKE 218/2018, 319/2021, 99/2022). Care was taken to minimize animal suffering and reduce the number of animals used in the experiments (3R policy)—the total number of mice used in our study was 429.

### 4.2. Neuropathic Pain Model

Mice were exposed to chronic constriction injury (CCI) of the sciatic nerve under isoflurane anesthesia, according to the procedure described by Bennett and Xie [105]. An incision was made below the right hipbone, and the biceps femoris and gluteus superficialis were separated. The sciatic nerve was exposed, and three ligatures (4/0 silk sutures) spaced 1 mm apart were tied loosely around the nerve distal to the sciatic notch until a brief twitch was elicited in the respective hind limb. CCI is a standard procedure that has been used in our laboratory for many years to induce neuropathic pain-related behavior in rodents [106,107,108]. On day 7 after surgery, all CCI-exposed mice fully developed tactile and thermal hypersensitivity compared to naïve animals as measured by the von Frey test (*p* < 0.0001) and the cold plate test (*p* < 0.0001) at all studied time points.

### 4.3. Behavioral Tests

#### 4.3.1. Von Frey Test

Tactile hypersensitivity was measured using calibrated nylon monofilaments of increasing strength (0.6 to 6 g; 6 g is the cutoff latency) (Stoelting, Wood Dale, IL, USA) to observe reactions to mechanical stimuli, as described previously [106]. The animals were placed in plastic cages with a wire mesh floor 5 min before the experiment, and von Frey filaments were applied to the midplantar surface of the right hind paw until the hind paw was lifted. In naïve animals, both hind paws were tested in the same way. This is a test used regularly in our laboratory [107,109,110].

#### 4.3.2. Cold Plate Test

Thermal hypersensitivity was assessed using a cold plate analgesia meter (Ugo Basile, Gemonio, Italy), as described previously [109]. The temperature of the cold plate was kept at 2 °C, and the cutoff latency was 30 s. The mice were placed on the cold plate separately, and the latency to lift the hind paw was recorded. In naïve animals, both hind paws were observed simultaneously. In CCI-exposed mice, the injured paw reacted first to the cold stimulus. This is a test used regularly in our laboratory [107,109,110].

### 4.4. Pharmacological Study

#### 4.4.1. Drugs and Routes of Administration

The following compounds were used in our experiments: fisetin (F; Cayman, Ann Arbor, MI, USA), peimine (P; Sigma‒Aldrich, St Louis, USA), artemisinin (Ar; Sigma‒Aldrich, St. Louis, MI, USA), astaxanthin (A; Sigma‒Aldrich, St Louis, USA), bardoxolone methyl (BM, Sigma‒Aldrich, St. Louis, MI, USA), 740 Y-P (7; Med Chem Express, Monmouth Junction, NJ, USA), morphine hydrochloride (M; Fagron, Krakow, Poland), buprenorphine (B; Polfa S.A., Warsaw, Poland) and oxycodone hydrochloride (O; Molteni Farmaceutici, Scandicci, Italy). All of these substances were administered via intrathecal injection (i.t.), a standard procedure in our laboratory [109,111]. It is performed by using a Hamilton syringe with a thin needle in accordance with instructions described previously [112]. The injections were performed in the lumbar segment of the spinal cord (between the L5 and L6 vertebrae) with a volume of 5 µL, and the tail reflex was an indicator of correct administration.

#### 4.4.2. Dose-Dependence Study

All intracellular pathway modulators were dissolved in 60% DMSO (reconstituted in water for injection) (Sigma‒Aldrich, St. Louis, MI, USA) and administered i.t. Artemisinin at doses of 1, 5, and 10 µg/5 µL; peimine at doses of 10, 20, and 60 µg/5 µL; fisetin at doses of 20, 60, 120, and 240 µg/5 µL; astaxanthin at doses of 0.5, 1, and 2 µg/5 µL; bardoxolone methyl at doses of 5, 10 and 20 µg/5 µL; and 740 Y-P at doses of 1, 5, 10, and 20 µg/5 µL were each administered on day 7 after CCI. Behavioral tests were performed 0.5, 1.5, 3, 5 and 24 h after injections in each case. The time points were selected based on our previous behavioral studies.

#### 4.4.3. Administration with Opioids

Based on the results of the dose-dependence study, we selected doses of each substance for combined administration with opioids. On day 7 after CCI, single i.t. injections of fisetin (60 µg/5 µL), peimine (20 µg/5 µL), astaxanthin (1 µg/5 µL), bardoxolone methyl (10 µg/5 µL) or 740 Y-P (10 µg/5 µL) were performed. Then, after 1 h, the vehicle- and intracellular pathway modulator-treated mice received a single i.t. injection of morphine (2.5 µg/5 µL), buprenorphine (1 µg/5 µL), oxycodone (1 µg/5 µL) or control (water for injections). Each pharmacological tool was combined with each of the opioid drugs tested in the experiment. Behavioral tests were conducted 0.5 h after opioid injections and 1.5 h after the first administration.

### 4.5. Data Analysis

The behavioral data are presented as means ± SEM in grams and seconds. One-way analysis of variance (ANOVA) was used to evaluate the experimental results, followed by Bonferroni’s post hoc test of selected pairs measured separately at each time point. The results from Figure 1, Figure 2 and Figure 3 were additionally evaluated using two-way ANOVA to detect time–drug interactions. All of the statistical analyses were performed using Prism (version 8.1.1 (330), GraphPad Software, Inc., San Diego, CA, USA). Additionally, for comparison of the results obtained 1.5 h after drug administration, calculations of ED_50_ and % MPE were performed. The Litchfield and Wilcoxon method was used to determine the antinociceptive dose necessary to produce a 50% response (ED_50_) and the 95% confidence interval on the quantitative data, which was automatically calculated with Pharm/PCS software, version 4. The percentage of the maximal possible antinociceptive effect (%MPE ± SEM) was calculated according to the following equation: %MPE = [(TL − BL)/(CUT − OFF − BL)] × 100%, where BL was the baseline latency and TL was the latency obtained after drug injection. The differences were considered significant when *p* < 0.05.

## 5. Conclusions

In summary, in our opinion, the inhibitors of the intracellular pathways studied here bring satisfactory results, because they are multitarget substances directed at several factors that were previously implicated to be important for nociceptive processes. In our opinion, modern polypharmacology should include combinations of drugs with a broad spectrum of action. Our research results indicate that substances such as peimine (MAPK and NFκB inhibitor) and astaxanthin (MAPK inhibitor, Nrf2 activator), apart from their pain-relieving properties, also effectively enhance opioid analgesia in neuropathy. Undoubtedly, astaxanthin is one of the most promising substances, but the selective activator of Nrf2, bardoxolone methyl, seems to be worthy of further experiments. The results of our research demonstrate how important it is to test multitarget substances, especially those that are already broadly used as dietary supplements, because they are safe for human consumption. The modulators of intracellular pathways studied here, especially astaxanthin, bring promising results for the development of new, more effective therapeutic interventions, and continuation of this research will broaden our knowledge regarding the mechanisms of neuropathy.

## Figures and Tables

**Table 1:** Calculated ED_50_ values for the effect of each of i.t. administered substances (artemisinin, 1, 5, 10 µg; fisetin, 20, 60, 120 µg; peimine, 10, 20, 60 µg; astaxanthin, 0.5, 1, 2 µg; bardoxolone methyl, 5, 10, 20 µg; 740 Y-P, 1, 5, 10 µg) with 95% confidence intervals in CCI-exposed mice measured by von Frey and cold plate tests 1.5 h after drug injection.

ED_50_	Von Frey Test	Cold Plate Test
artemisinin	-	-
fisetin	**68.5** (27.3 ± 171.5)	**40.5** (14.7 ± 111.3)
peimine	**214.2** (19.8 ± 2321.5)	**60.5** (14.3 ± 256.6)
astaxanthin	**0.7** (0.2 ± 1.9)	**0.6** (0.4 ± 0.8)
bardoxolone methyl	**22.0** (4.9 ± 98.4)	**4.8** (2.5 ± 9.1)
740 Y-P	**0.7** (20.4 ± 200.6)	**7.5** (2.0 ± 27.9)

**Table 2:** Calculated %MPE values for the highest dose of each tested substances used in our experiments in CCI-exposed mice in von Frey and cold plate tests, 1.5 h after drug administration. * *p* < 0.05, ** *p* < 0.01 and *** *p* < 0.001 indicate differences vs. V-treated CCI-exposed mice.

%MPE	Von Frey Test	Cold Plate Test
vehicle	−0.22 ± 1.99	0.76 ± 4.12
artemisinin (10 µg/5 µL)	3.38 ± 8.32	−10.34 ± 4.83
fisetin (240 µg/5 µL)	71.57 ± 12.07 ***	41.31 ± 5.79 ***
peimine (60 µg/5 µL)	30.91 ± 10.73 *	22.76 ± 2.65 **
astaxanthin (2 µg/5 µL)	64.82 ± 10.14 ***	71.05 ± 6.14 ***
bardoxolone methyl (20 µg/5 µL)	42.96 ± 8.77 **	67.85 ± 2.10 ***
740 Y-P (20 µg/5 µL)	38.64 ± 9.93 **	38.30 ± 13.14 ***

## Data Availability

The data presented in this study are available on request from the corresponding author.
